# The impact of neoadjuvant concurrent chemoradiation on exosomal markers (CD63 and CD9) expression and their prognostic significance in patients with rectal adenocarcinoma

**DOI:** 10.18632/oncotarget.28025

**Published:** 2021-07-20

**Authors:** Moh’d Khushman, Pranitha Prodduturvar, Wadad Mneimneh, Valeria Dal Zotto, Shalla Akbar, Leander Grimm, Paul Rider, John Hunter, Omar Alkharabsheh, Girijesh Kumar Patel, Jesus C. Fabregas, Ajay P. Singh

**Affiliations:** ^1^Hematology-Oncology, O’Neal Comprehensive Cancer Center, The University of Alabama at Birmingham, Birmingham, AL, USA; ^2^Medical Oncology, Mayo Clinic, Rochester, MN, USA; ^3^Pathology, Case Western Reserve University, Cleveland, OH, USA; ^4^Pathology, The University of South Alabama, Mobile, AL, USA; ^5^Colorectal Surgery, The University of South Alabama, Mobile, AL, USA; ^6^Department of Cell Biology and Biochemistry, Texas Tech University Health Sciences Center, Lubbock, TX, USA; ^7^Harvard T.H. Chan School of Public Health, Boston, MA, USA

**Keywords:** exosomes, exosomal markers, CD63, CD9, neoadjuvant chemoradiation

## Abstract

Introduction: Exosomes have pivotal roles in cancer development. The impact of neoadjuvant concurrent chemoradiation (NCCR) on exosomal markers (CD63 and CD9) expression and their prognostic significance in patients with rectal adenocarcinoma are yet to be explored.

Materials and Methods: Between 2015 and 2018, 33 patients had rectal adenocarcinoma treated with NCCR and had pre-NCCR biopsy and post-NCCR resected rectum. CD63 and CD9 expression was assessed by immunohistochemistry (IHC). The short-term surrogate endpoint neoadjuvant rectal (NAR) score was used for assessment of prognostic significance. Un-Paired *t*-test was used for statistical analysis.

Results: The mean tumor CD63 and CD9 scores in pre-NCCR biopsy vs. post-NCCR resected rectum were 106 vs. 165 (*P* = 0.0022) and 136 vs. 215 (*P* < 0.0001) respectively. The mean tumor CD63 and CD9 scores respectively in pre-NCCR biopsy was 99 and 130 in patients with low-intermediate NAR score compared to 117 and 144 in patients with high NAR score (*P* = 0.4934) (*P* = 0.5519). The mean tumor CD63 and CD9 scores respectively in post-NCCR resected rectum was 155 and 205 in patients with low-intermediate NAR score compared to 180 and 230 in patients with high NAR score (*P* = 0.3793) (*P* = 0.2837).

Conclusions: The expression of the exosomal markers (CD63 and CD9) increased in patients with rectal adenocarcinoma after treatment with NCCR. The exosomal markers (CD63 and CD9) may have a prognostic significance. There was a trend for higher CD63 and CD9 expression in patients with high NAR score compared with low-intermediate NAR scores. The lack of statistical significance is likely due to the small sample size.

## INTRODUCTION

Colorectal cancer (CRC) is a common disease. In 2021, an estimated 149,500 new cases of CRC and 52,980 of CRC-related deaths are expected [1]. The treatment of locally advanced rectal cancer is different from colon cancer. Surgical resection is the cornerstone treatment modality following a course of neoadjuvant therapy [2, 3]. Tumor response to neoadjuvant therapy may predict long term outcomes such as disease-free survival (DFS) and overall survival (OS) [4].

Exosomes are a subset of extracellular vesicles that are 40–100 nm in diameter [5]. Exosomal membranes are enriched in endosome-specific tetraspanins (CD9, CD63 and CD81) [6]. Exosomes are functional nanocarriers of a complex cargo of proteins, lipids, and nucleic acids and transfer it between the donor and recipient cells. Thus, they represent a novel mode of intercellular communication playing important roles in tumor progression, metastasis, chemotherapy resistance and immune response [5, 7–14]. Exosomes and their biologically active cargo differ between different cells and may offer prognostic information [14–16].

During treatment with chemotherapy and/or radiotherapy, exosomes and their content undergo different changes that play a role in tumor characteristics. Radiation-induced changes in exosomes promote migration of recipient cells and may facilitate progression during radiotherapy [17]. Radiation-derived exosomes promoted proliferation and helped recipient cancer cells to survive radiation *in vitro* and decreased the survival of tumor-bearing mice *in vivo* [18]. Chemotherapy-induced exosomes have been shown to carry different cargo loads compared to no-chemotherapy-induced exosomes. Apart from chemoresistance, there is growing evidence to show that chemotherapy-induced exosomes influence tumor behavior, metastasis and immune response [10, 19].

Neoadjuvant rectal (NAR) score is a short-term surrogate endpoint for predicting long-term endpoints such as OS. Calculating the NAR score is performed using tumor variables before and after treatment with neoadjuvant therapy including the clinical T (cT), the pathological T (pT) and pathological N (pN) stages using the formula ([5 ypN − 3 (cT − ypT) + 12]^2^/9.61) [20, 21]. The NAR score was validated using the patient dataset from NSABP R-04 clinical trial where NAR scores were categorized as low, intermediate, and high. Low (NAR < 8), intermediate (NAR = 8–16) and high (NAR > 16) scores were associated with OS (*p* = 0.0001) with 5-year OS of 92, 89 and 68% respectively [22].

The impact of NCCR on CD63 and CD9 expression and their prognostic significance in patients with rectal adenocarcinoma is yet to be explored. In this study, we explored the impact of NCCR on CD63 and CD9 expression pattern and their prognostic significance using the short-term surrogate endpoint NAR score.

## RESULTS

### Patients baseline characteristics

Our cohort (*N* = 33) included patients identified between 2015 and 2018, with rectal adenocarcinoma treated with NCCR and had pre-NCCR biopsy and post-NCCR resected rectum examined for exosomal markers expression using IHC. Patients’ baseline characteristics are summarized in Table 1. The Median age was 59 years (range 34–71). Caucasians, African Americans and other ethnicities represented 23 (70%), 9 (27%) and 1 (3%) patients respectively. Males and Females represented 26 (79%) and 7 (21%) patients respectively. The primary tumor stage as assessed by pelvic magnetic resonance imaging (MRI) was cT1, cT2, cT3 and cT4 in 0 (0%), 2 (6%), 29 (88%) and 2 (6%) patients respectively. The lymph node stage as assessed by pelvic MRI was cN0, cN1 and cN2 in 10 (30%), 12 (36%) and 11 (33%) patients respectively. All but 3 (9%) patients had no evidence of distant metastasis. In the patients with distant metastasis, the site of metastasis was liver in 2 patients and peritoneum in 1 patient. Patients with low, intermediate and high NAR scores were 2 (6%), 17 (52%) and 14 (42%) patients respectively.

**Table 1:** Patients baseline characteristics (*N* = 33)

**Age (Years)**	
Median (Range)	59 (34–71)
**Sex**	***N* (%) **
Male	26 (79%)
Female	7 (21%)
**Ethnicity**	***N* (%) **
White	23 (70%)
African American	9 (27%)
Other	1 (3%)
**Stage**	***N* (%) **
**cT stage**	
cT1	0 (0%)
cT2	2 (6%)
cT3	29 (88%)
cT4	2 (6%)
**cN stage**	
cN0	10 (30%)
cN1	12 (36%)
cN2	11 (33%)
**cM stage**	
cM0	30 (91%)
cM1	3 (9%)
**NAR score**	***N* (%) **
Low (<8)	2 (6%)
Intermediate (= 8–16)	17 (52%)
High (>16)	14 (42%)

### Expression of CD63 and CD9 using immunohistochemistry

**Figure 1:**
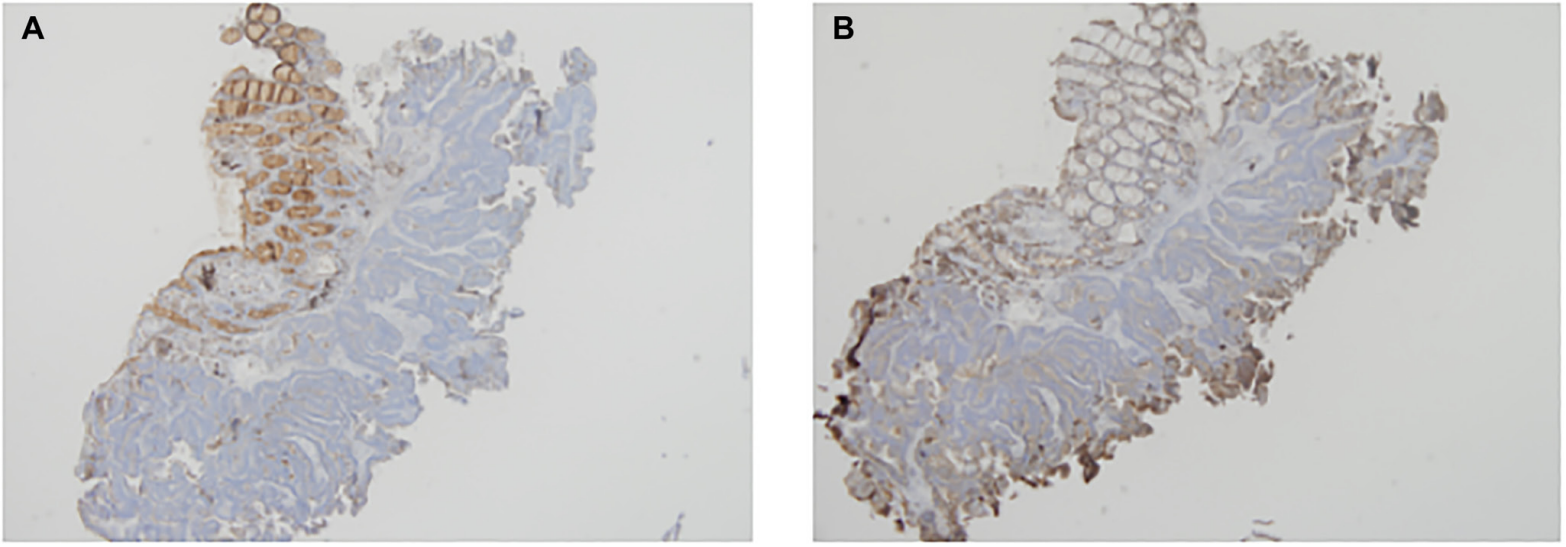
The expression of CD63 and CD9 using IHC in the pre-NCCR biopsy is shown in (**A**) and (**B**) respectively.

**Figure 2:**
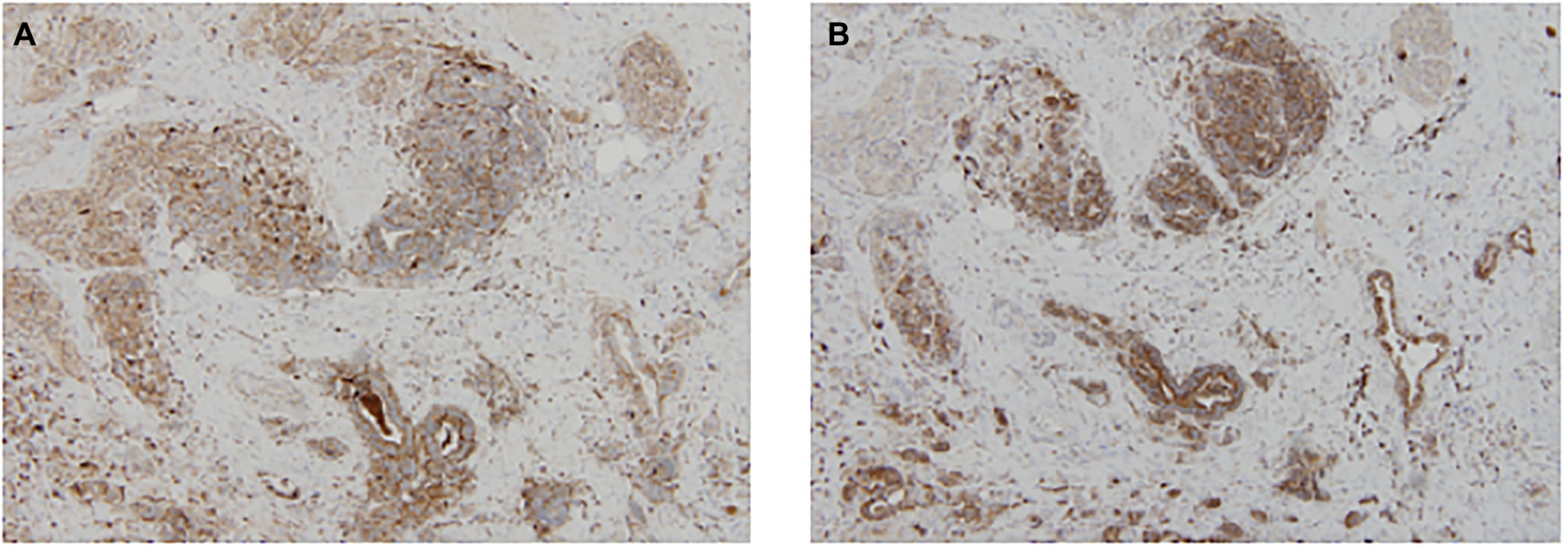
The expression of CD63 and CD9 using IHC in the post-NCCR resected rectum is shown in (**A**) and (**B**) respectively.

The expression of CD63 and CD9 using IHC in the pre-NCCR biopsy is shown in Figure 1A and 1B respectively. The expression of CD63 and CD9 using IHC in the post-NCCR resected rectum is shown in Figure 2A and 2B respectively.

### Tumor exosomal markers (CD63 and CD9) expression scores

**Figure 3:**
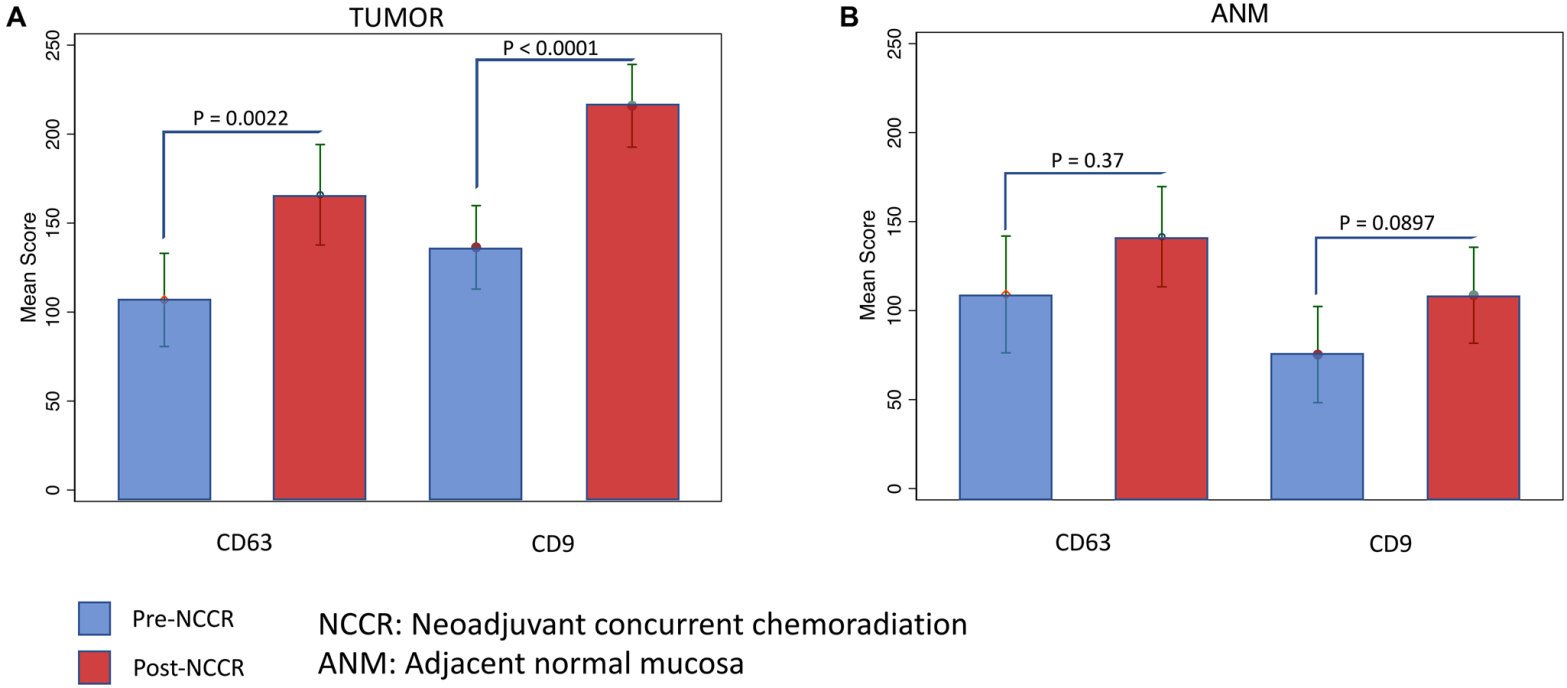
The differences in the median tumor and ANM CD63 and CD9 scores between pre-NCCR rectal biopsy and post-NCCR resected rectum is visualized in (**A**) and (**B**) respectively.

The mean tumor CD63 score in pre-NCCR rectal biopsy vs. post-NCCR resected rectum was 106 vs. 165 (*P* = 0.0022). The mean tumor CD9 score in pre-NCCR rectal biopsy vs. post-NCCR resected rectum was 136 vs. 215 (*p* < 0.0001). The mean tumor scores for CD63 and CD9 in pre-NCCR rectal biopsy vs. post-NCCR resected rectum are summarized in Table 2. The differences in the median tumor CD63 and CD9 scores between pre-NCCR rectal biopsy and post-NCCR resected rectum is visualized in Figure 3A.

**Table 2:** The mean tumor and adjacent normal mucosa scores for CD63 and CD9 in pre-NCCR rectal biopsy vs. post-NCCR resected rectum

Tissue	Number	CD63 Mean score Median (SD)	CD9 Mean score Median (SD)
**Tumor**			
Pre-NCCR Rectal Biopsy	33	106 (73.9)	136 (66.1)
Post-NCCR Resected Rectum	33	165 (79.6)	215 (65.5)
		(*P* = 0.0022)	(*P* < 0.0001)
**Adjacent Normal Mucosa**			
Pre-NCCR Rectal Biopsy	16	166 (92.5)	104 (76.2)
Post-NCCR Resected Rectum	16	183 (79.4)	145 (76.1)
		(*P* = 0.37)	(*P* = 0.0897)

Abbreviations: NCCR, Neoadjuvant concurrent chemoradiation; SD, Standard deviation.

### Adjacent normal mucosa exosomal markers (CD63 and CD9) expression scores

The exosomal markers expression scoring in the ANM from pre-NCCR rectal biopsy and post-NCCR resected rectum was only performed in 16 out of 33 patients (due to ANM tissue availability). The mean ANM CD63 score in pre-NCCR rectal biopsy vs. post-NCCR resected rectum was 166 vs. 183 (*p* = 0.37). The mean tumor CD9 score in pre-NCCR rectal biopsy vs. post-NCCR resected rectum was 104 vs. 145 (*p* = 0.0897). The mean ANM scores for CD63 and CD9 in pre-NCCR rectal biopsy vs. post-NCCR resected rectum are summarized in Table 2. The differences in the median ANM CD63 and CD9 scores between pre-NCCR rectal biopsy and post-NCCR resected rectum is visualized in Figure 3B.

### The prognostic value of tumor exosomal markers (CD63 and CD9)

Only 2 patients had low NAR score, and thus low and intermediate NAR scores were grouped together. The tumor pre-NCCR rectal biopsy mean CD63 score was 99 in patients with low-intermediate NAR scores compared to 117 in patients with high NAR scores (*P* = 0.4934). The tumor post-NCCR resected rectum mean CD63 score was 155 in patients with low-intermediate NAR scores compared to 180 in patients with high NAR scores (*P* = 0.3793). The tumor pre-NCCR rectal biopsy mean CD9 score was 130 in patients with low-intermediate NAR scores compared to 144 in patients with high NAR scores (*P* = 0.5519). The tumor post-NCCR resected rectum mean CD9 score was 205 in patients with low-intermediate NAR scores compared to 230 in patients with high NAR scores (*P* = 0.2837). The mean tumor scores for CD63 and CD9 in pre-NCCR rectal biopsy and post-NCCR resected rectum according to NAR score are summarized in Table 3.

**Table 3:** The mean tumor scores for CD63 and CD9 in pre NCCR rectal biopsy and post NCCR resected rectum according to NAR score

Neoadjuvant Rectal Score	Number	Tumor Pre-NCCR Mean score	Tumor Post-NCCR Mean score
Exosomal Marker CD63			
Low-Intermediate (NAR ≤ 16)	19	99	155
High (NAR > 16)	14	117	180
		(*P* = 0.4934)	(*P* < 0.3793)
Exosomal Marker CD9			
Low-Intermediate (NAR ≤ 16)	19	130	205
High (NAR > 16)	14	144	230
		(*P* = 0.5519)	(*P* = 0.2837)

## DISCUSSION

Exploring exosomes and the mechanisms that govern their generation and functions is motivated by their potential as diagnostic, therapeutic and prognostic tools in many diseases especially cancer [16, 23]. Increasingly, exosomes research is aimed at isolation methods, understanding their diverse range of biological functions and exploring their local and distant interactions [24–26]. Current characterization of biological activities of exosomes in cancer has largely been carried out on cell lines and animal models and therefore it remains unclear if some of the reported properties and functions of exosomes will be reproduced in blood or tumor specimens [27, 28].

During treatment with radiotherapy, exosomes and their content undergo different changes that play a role in tumor characteristics. Exosomes isolated from irradiated donor cells boost the motility of the head and neck squamous cell carcinoma (HNSCC) cells BHY and FaDu through enhanced AKT-signaling. That promoted migration of HNSCC cells is potentially driving HNSCC progression during treatment with radiotherapy [17]. In STS26T human malignant peripheral nerve sheath tumor cells, U87 Glioma cells and SH-SY5Y human neuroblastoma cells, radiation-derived exosomes increased proliferation and enabled recipient cancer cells to survive radiation *in vitro*. Moreover, radiation-derived exosomes increased tumor burden and decreased survival in an *in vivo* model [18]. In breast cancer cells (MCF-7), exosome biogenesis and secretion could be activated by X-ray in a dose-dependent fashion suggesting the therapeutic response of cells via ROS and exosome activity [29]. Chemotherapy-induced exosomes have been shown to carry different cargo loads compared to no-chemotherapy-induced exosomes [10]. Apart from mediating chemotherapy resistance, there is growing evidence to show that chemotherapy-induced exosomes influence tumor behavior, metastasis and immune response. [10, 19] Research efforts to explore the impact of chemotherapy concurrent with radiotherapy on exosomes are needed, and patients with rectal adenocarcinoma is a group of patients that will likely benefit from such efforts.

In our patients with rectal cancer, a differential expression of exosomal markers was observed between tumor tissue and ANM in both pre-NCCR rectal biopsies and post-NCCR resected rectum specimens (Table 2). Exposure to NCCR resulted in an increase in the tumor exosomal markers (CD63 and CD9) expression in the post-NCCR resected rectum compared to pre-NCCR rectal biopsies. There was a trend toward increased expression of ANM exosomal markers (CD63 and CD9) in the post-NCCR resected rectum compared to pre-NCCR rectal biopsies but it didn’t reach statistical significance (probably due to small sample size, *N* = 16). To our knowledge, our study is the first to show that exposure to NCCR increases the expression of CD63 and CD9 in patients with rectal cancer using IHC. This observation is suggestive of a possible role of exosomes in the adaptive response to NCCR in the tumor and may be the microenvironment.

Exosomal markers (CD63 and CD9) expression and its prognostic significance have been explored in different tumors. Decreased expression of CD63 and CD9 was associated with metastatic potential in patients with breast cancer, colon cancer, pancreatic cancer and Melanoma [16, 30–33]. Low expression of CD63 and CD9 was associated with poor prognosis in patients with breast cancer, pancreatic cancer, and lung cancer [16, 33, 34]. Increased CD63 expression was associated with poor prognosis in patients with gastrointestinal stromal tumors [35].

The design of clinical trials in patients with rectal adenocarcinoma has often used long-term endpoints such as DFS and OS. Unfortunately, this has slowed our progress due to the time needed for long-term outcomes to take place and for the clinical trials results to be reported. Incorporating short-term surrogate endpoints for DFS and OS such as NAR score in clinical trials design is expected to help with earlier assessment of the positive or negative interventions [20, 22].

Our work showed that the expression of CD63 and CD9 in pre-NCCR rectal biopsies and post-NCCR resected rectum specimens may have a prognostic significance. In both pre-NCCR rectal biopsies and post-NCCR resected rectum, patients with high NAR (>16) had higher CD9 and CD63 scores compared to patients with low (< 8) and intermediate NAR (8–16) scores. The lack of statistical significance is likely due to the small sample size. Overall, patients with higher CD63 and CD9 scores may have high NAR scores and worse prognoses. However, patients with lower CD63 and CD9 scores may have low-intermediate NAR scores and a better prognosis. To our knowledge, this is the first study to explore the expression pattern and prognostic significance of CD63 and CD9 in patients with rectal cancer treated with NCCR using IHC.

This study has several limitations. It included small cohort (*N* = 33) from a single academic cancer center. This is also a retrospective study that is prone to selection bias and provides inferior level of evidence compared to prospective studies. Moreover, the method of exosomal markers (CD63 and CD9) detection used in this study is IHC. Apart from providing data about exosomal markers expression, IHC staining doesn’t provide data about the origin of exosomes and their function and content. Despite the demonstrated impact of concurrent chemotherapy and radiation on the expression of CD63 and CD9 and their possible prognostic significance, our results and conclusion should be interpreted with caution and rather considered hypothesis-generating. Studies that can address our limitations and of larger cohorts need to be conducted to confirm the results and explore the underlying mechanisms.

## MATERIALS AND METHODS

### Rectal cancer tissue collection

This is a retrospective study. Our cohort included patients with rectal adenocarcinoma treated with NCCR and had pre-NCCR biopsy and post-NCCR resected rectum examined for exosomal markers expression using immunohistochemistry (IHC). Patients were identified from the colorectal cancer database. This study was approved by the Institutional Review Board (IRB).

### Immunohistochemical staining

Five μm sections were obtained from every rectal cancer biopsy and resected rectal cancer specimen. Two were obtained from the tumor and two were obtained from the adjacent normal mucosa (ANM). To study the expression pattern of the exosomal markers (CD63 and CD9), immunohistochemical staining was performed.

### Scoring of CD63 and CD9 expression

The scoring of CD63 and CD9 staining was carried out by two pathologists. They, independently, graded the intensity of cytoplasmic staining from 1 to 3: 1 (weak), 2 (moderate) and 3 (strong) and the percentage of stained cells on each section in 10% increments. A multiplicative score was calculated for each tissue section by multiplying the percentage of positive cells by the intensity of the staining [36]. The average score between the two pathologists was calculated for each section.

### Neoadjuvant rectal score calculation

Calculating the NAR score was performed using tumor variables before and after treatment with neoadjuvant therapy including the clinical T (cT), the pathological T (pT) and pathological N (pN) stages using the formula ([5 ypN − 3 (cT − ypT) + 12]^2^/9.61). The NAR scores were categorized as low (NAR < 8), intermediate (NAR = 8–16), and high (NAR > 16). Because only two patients had low NAR score, patients with low and intermediate NAR scores were grouped together.

### Statistical analysis

The difference in CD63 and CD9 expression between pre-NCCR biopsies of the tumor/ANM and post-NCCR resected rectal specimens/ANM was compared using unpaired *t*-test. The difference in tumor CD63 and CD9 expression between low-intermediate and high NAR scores was compared using an unpaired *t*-test. Statistical significance was defined as *p* < 0.05, and all tests were two-sided. Tests were performed using GraphPad Prism version 9, GraphPad Software (San Diego, CA, USA), https://www.graphpad.com.

## CONCLUSIONS

Using IHC, the exosomal markers (CD63 and CD9) expression increased in patients with rectal adenocarcinoma after treatment with NCCR and thus suggest a possible role of these exosomes in adaptive response to NCCR. Further follow-up and laboratory studies are required to precisely understand the underlying mechanism(s). The exosomal markers (CD63 and CD9) may have a prognostic significance. There was a trend for higher CD63 and CD9 expression in patients with high NAR score compared with low-intermediate NAR score. The lack of statistical significance is likely due to small sample size.

## Abbreviations

NCCR: Neoadjuvant Concurrent Chemoradiation
IHC: Immunohistochemistry
ANM: Adjacent Normal Mucosa
CRC: Colorectal Cancer
DFS: Disease Free Survival
OS: Overall Survival
EVs: Extracellular Vesicles
IRB: Institutional Review Board
Q-score: Quick-score
HNSCC: Head and Neck Squamous Cell Carcinoma
MRI: Magnetic Resonance Imaging
NAR Score: Neoadjuvant Rectal Score
